# Comparison of volume‐ and surface‐based magnetic resonance imaging morphometry algorithms in the detection of focal cortical dysplasia

**DOI:** 10.1002/epi.70326

**Published:** 2026-06-03

**Authors:** Lara Bücheler, Marco Reisert, Theo Demerath, Florian Mayer, Christian Moeller, Joshua Reß, Yiwen Li Hegner, Dirk‐Matthias Altenmüller, Victoria San Antonio‐Arce, Christian Scheiwe, Mukesch Shah, Jürgen Beck, Soroush Doostkam, Thomas Bast, Bernhard J. Steinhoff, Andreas Schulze‐Bonhage, Horst Urbach, Marcel Heers

**Affiliations:** ^1^ Epilepsy Center, Medical Center, member of the European Reference Network for Rare and Complex Epilepsies EpiCARE, Faculty of Medicine University of Freiburg Freiburg Germany; ^2^ Department of Neuroradiology, Faculty of Medicine University of Freiburg Freiburg Germany; ^3^ Department of Radiology, Medical Physics, Faculty of Medicine University of Freiburg Freiburg Germany; ^4^ Department of Pediatrics and Adolescent Medicine Medical University of Vienna, member of European Reference Network for Rare and Complex Epilepsies EpiCARE Vienna Austria; ^5^ Neurocenter, IT‐Service, Faculty of Medicine University of Freiburg Freiburg Germany; ^6^ Department of Neurology and Epileptology, member of European Reference Network for Rare and Complex Epilepsies EpiCARE, Hertie Institute for Clinical Brain Research University of Tübingen Tübingen Germany; ^7^ Department of Neurosurgery, Faculty of Medicine University of Freiburg Freiburg Germany; ^8^ Institute of Neuropathology, Faculty of Medicine University of Freiburg Freiburg Germany; ^9^ Epilepsy Center Kork Kehl‐Kork Germany

**Keywords:** automated lesion detection, epilepsy surgery, focal cortical dysplasia, machine learning, MRI morphometry

## Abstract

**Objective:**

Detection of focal cortical dysplasia (FCD) remains a major challenge in presurgical epilepsy diagnostics. Magnetic resonance imaging (MRI) morphometry increasingly improves lesion detection and postsurgical outcomes. The volume‐based Morphometric Analysis Program, version 2018 (MAP18) with integrated artificial neural network and surface‐based Multi‐centre Epilepsy Lesion Detection (MELD; MELD Graph) MRI morphometry algorithms have demonstrated diagnostic utility, but their performance has not been directly compared within a single cohort of surgically treated patients with histopathologically confirmed FCD.

**Methods:**

We retrospectively analyzed 3‐T MRIs of 32 FCD patients who underwent epilepsy surgery. MAP18 was applied to MP2RAGE (magnetization‐prepared 2 rapid gradient echo) sequences and MELD to T1MPRAGE (magnetization‐prepared rapid gradient echo) and three‐dimensional FLAIR (fluid‐attenuated inversion recovery) sequences. Binary lesion masks served as ground truth for evaluating detection accuracy, precision, and recall at cluster and voxel levels. Clinical correlation analysis assessed spatial concordance with seizure onset zone (SOZ) and irritative zone (IZ) from video‐electroencephalography (EEG) and postoperative outcome.

**Results:**

The cohort included 34 histopathologically proven FCD lesions (91.2% FCD II, 5.8% mild malformation of cortical development, 2.9% unspecified). MELD identified 32 of 34 lesions (94.1%), and MAP18 identified 33 of 34 lesions (97.1%), with concordance in 32 of 34 lesions (94.1%). At the cluster level, MELD showed significantly higher precision (*p* < .001) and fewer false positive clusters per patient (median 0 vs. 3, *p* < .001). Voxelwise analysis revealed that MELD demonstrated significantly higher precision than MAP18 (.42 vs. .13, *p* < .0001) and recall (.44 vs. .23, *p* < .01). Lobar concordance with SOZ reached 90.6% and with IZ reached 87.5%. Among invasive EEG patients, overlap with SOZ and IZ was 100%. Seizure freedom (Engel I) was achieved in 87.5% of patients.

**Significance:**

Under standardized high‐resolution 3‐T MRI, both algorithms achieved high detection rates with strong concordance. Combined application may enhance presurgical lesion detection, with results limited to surgically treated patients.

**Key points**

- MELD and MAP18 achieved 94%–97% detection rates for focal cortical dysplasia using standardized 3‐T imaging protocols.
- MELD demonstrated significantly higher precision (*p* < .0001) and recall (*p* < .01) than MAP18.
- Both algorithms showed 90% lobar concordance with seizure onset zone in scalp EEG and 100% overlap in invasive EEG.
- Eighty‐eight percent of patients achieved seizure freedom.

## 1 INTRODUCTION

Focal epilepsy often begins early in childhood or adolescence and remains refractory to pharmacological treatment in a substantial proportion of patients with focal cortical dysplasia (FCD).^1^ Drug‐resistant epilepsy is defined by the persistence of epileptic seizures despite treatment with at least two appropriately selected, well‐tolerated, and optimally dosed antiseizure medications.^2^ For these patients, surgical resection of the epileptogenic zone offers an effective alternative, achieving seizure freedom rates of approximately 60%–70% following removal of the identified epileptogenic zone, although outcomes remain worse in individuals with nonlesional epilepsy.^3^

Complete resection of FCDs based on magnetic resonance imaging (MRI) criteria is a positive predictor of postsurgical outcome.^4^ However, preoperative detection and precise localization of FCDs remain major clinical challenges. Conventional visual assessment of structural MRI sequences often demonstrates inadequate sensitivity,^5^ particularly in patients with subtle findings, thereby complicating surgical planning and adversely impacting postoperative outcomes.^6^

To address these limitations, MRI postprocessing methods are increasingly deployed for FCD detection, with the potential to significantly improve presurgical evaluation and patient selection for epilepsy surgery. These automated approaches leverage machine learning to identify subtle structural abnormalities that may escape visual detection. The most widely used techniques employ either volume‐based or surface‐based classifiers that analyze multiple imaging features.

The volume‐based Morphometric Analysis Program, version 2018 (MAP18) compares individual patient scans to a normative database using statistical maps of the gray–white matter junction, cortical thickness, and signal intensity. These parameters are combined in an artificial neural network (ANN), which achieved a sensitivity of 81% and a specificity of 84% for detecting FCDs in an external validation dataset.^7^ In the present study, MAP18 was used with the ANN enabled. Sensitivity can be further enhanced by analyzing high‐contrast magnetization‐prepared 2 rapid gradient echo (MP2RAGE) MRI sequences instead of magnetization‐prepared rapid gradient echo (T1MPRAGE) sequences.^8^

The Multi‐centre Epilepsy Lesion Detection (MELD) project developed a surface‐based deep learning classifier using data from more than 1000 patients across 23 international epilepsy centers.^9^, ^10^ Building on this foundation, the MELD Graph implemented a graph neural network (GNN) architecture that treats each cortical vertex as a node, integrating morphological information from neighboring vertices to exploit cortical topology. Analyzing surface features including cortical thickness, curvature, and fluid‐attenuated inversion recovery (FLAIR) intensity, MELD Graph operates in a fully automated manner. In a multicenter validation cohort of 703 patients with FCD and 482 controls across 23 epilepsy centers, MELD Graph achieved a sensitivity of 72% and a positive predictive value (PPV) of 76% in a site‐independent test cohort, substantially outperforming the prior algorithm (PPV = 46%).^10^

Although both MAP18 and MELD have demonstrated value in diagnosing FCD, no direct comparison of their performance has been conducted within a single cohort of surgically treated patients with histopathologically confirmed FCD. Such a comparison is critically needed to guide clinical implementation and inform centers deciding which automated detection tool to adopt. Moreover, understanding the complementary strengths of these methods could optimize their clinical integration.

In this retrospective study, we conducted a head‐to‐head comparison of MAP18 and MELD detection performance using standardized, harmonized high‐resolution imaging protocols (T1MPRAGE, MP2RAGE, and three‐dimensional [3D] FLAIR sequences). Our analysis addressed three key objectives. First, we quantified the performance of each algorithm using cluster‐based metrics: overall success rate, recall, precision, F1‐score, and false positive rate. Second, we investigated spatial concordance between the algorithms, examining whether regions detected by both methods corresponded to more reliable and clinically validated FCD areas. Third, we descriptively evaluated the spatial relationship between the histopathologically confirmed FCDs and clinical markers—specifically, the electroencephalographic seizure onset zone (SOZ) and irritative zone (IZ)—as well as postoperative seizure outcomes, to characterize the clinical context of detected lesions.

## 2 MATERIALS AND METHODS

### 2.1 Study design and patient selection

Patients in this study were retrospectively selected from records at the Epilepsy Center, University Medical Center Freiburg. Eligibility was determined based on prior internal presurgical diagnostics and subsequent surgical resection, with histopathological confirmation of FCD type I, FCD type II, or mild malformation of cortical development (mMCD) between January 2018 and April 2024. Of 124 patients with histopathological diagnosis of FCD I, FCD II, or mMCD, 67 were excluded due to involvement of the temporal pole, 17 due to absence of MP2RAGE MRI sequences, and eight due to prior brain surgery or second resection, resulting in a final cohort of 32 patients with extratemporal lobe FCD. The exclusion of temporal pole lesions was implemented to exclude patients with gray–white matter blurring of the temporal pole^11^ and because postprocessing algorithms may exhibit different performance characteristics in these regions.^11^ Additionally, these lesions are still imperfectly characterized histopathologically, resulting in a low inter‐ and intraobserver reproducibility among neuropathologists.^12^, ^13^ Furthermore, a dedicated comparison of MELD and MAP18 in structural temporal lobe epilepsy has recently been reported.^14^

A flow diagram illustrating the patient screening process, exclusion criteria, and final cohort inclusion is provided in Figure S1. Detailed clinical characteristics of the included patients are summarized in Table 1. The study was conducted in accordance with the Declaration of Helsinki and received approval from the local ethics committee (University of Freiburg 23–1503‐S1‐retro). All participants provided written informed consent for the retrospective scientific use of their data obtained during presurgical epilepsy diagnostics.

**TABLE 1:** Patient characteristics.

Variable	Data
Number of patients/lesions	32/34 (1 patient, 3 FCDs [all resected])^a^
Sex, M/F	15 (47%)/17 (53%)
Age, years, mean ± SD (range)	21 ± 13 (1–53)
Epilepsy duration, years (IQR)	8.5 (2–18.5)
Histopathology	FCD II: 31 (91.2%), mMCD: 2 (5.8%), not specified: 1 (2.9%)
Lesion localization	Frontal: 19 (55.9%), temporal: 3 (8.9%), parietal: 2 (5.9%), central: 5 (14.7%), multilobar: 5 (14.7%)
Lesion volume, cm^3^, median (IQR)	5.0 (2.6–13.3)
Invasive EEG	11 (34%)
Follow‐up	31 patients with follow‐up of >12 months: median = 25 months (SD = 14)

*Note*: Data represent *n* (%) unless otherwise indicated.

Abbreviations: EEG, electroencephalography; F, female; FCD, focal cortical dysplasia; IQR, interquartile range; M, male; mMCD, mild malformation of cortical development.

^a^ Additionally, one patient with two FCDs based on magnetic resonance imaging criteria, both detected by both algorithms (only one FCD resected and histopathologically confirmed, patient seizure‐free).

### 2.2 MRI acquisition

All patients underwent a dedicated structural MRI of the brain following the protocol designed to reveal potentially epileptogenic lesions on a 3‐T scanner (Magnetom Prisma, Siemens) as part of the presurgical epilepsy workup.^15^ The imaging protocol was standardized across all subjects and included high‐resolution T1MPRAGE, MP2RAGE, and 3D FLAIR sequences with 1‐mm isotropic voxels. Images were visually inspected for quality and artifacts before further analysis. All datasets were stored in pseudoanonymized format and served as input for the MAP18 and MELD pipelines, which applied their integrated preprocessing steps to ensure consistent data normalization and registration on in‐house computers.

### 2.3 MELD classifier

The MELD classifier is an open‐source, surface‐based deep learning algorithm designed for the automated detection of FCDs (https://github.com/MELDProject/meld_graph). MELD integrates cortical surface reconstructions with morphometric and signal intensity features to compute subject‐specific lesion probabilities at the vertex level. Preprocessing was performed using FastSurfer v1.1.2 (https://deep‐mi.org/research/fastsurfer/)^16^ with FreeSurfer v7.2.0 (https://surfer.nmr.mgh.harvard.edu/pub/dist/freesurfer/7.2.0/)^17^ applied to coregistered T1MPRAGE and 3D FLAIR images.

Extracted features included cortical thickness, curvature, and gray–white matter boundary blurring, derived from T1 and FLAIR intensity sampled from both cortical and subcortical layers. The trained GNN outputs lesion probability maps, which are projected back from surface space onto each subject's native MRI. The output of this process includes lesion probability heatmaps, *z*‐scored individual feature maps, saliency maps that depict the features driving the network's predictions, and confidence scores.

The version of MELD used in this analysis (MELD Graph, version 2.2.4) is a trained GNN validated across multiple international epilepsy centers, demonstrating detection rates ranging from 61% to 81% across different imaging sites with 1.5‐ and 3‐T MRI.^10^, ^18^

### 2.4 MAP18 classifier

MAP18 is a volumetric postprocessing tool designed to detect subtle cortical abnormalities by comparing individual patient MRI data with a normative control database.^19^, ^20^ MAP18 is implemented within the SPM12 framework (Wellcome Centre for Human Neuroimaging, London, UK; https://www.fil.ion.ucl.ac.uk/spm/software/spm12/). MAP18 generates three morphometric contrasts: extension (gray matter into white matter), junction (gray–white matter blurring), and thickness maps.

Potential lesion clusters are identified using a shallow feedforward ANN integrated into MAP18. This ANN combines morphometric features from extension, junction, and thickness maps to generate FCD probability maps. MAP18 processing was performed using SPM12 running on MATLAB R2019a on a local workstation. All procedures, such as formal data conversion (DICOM to NifTI) and inverse normalization into native space, were systematically documented to ensure reproducibility.

According to the methodology of Demerath et al., lesion clusters were defined using a minimum volume threshold of .23 mL, optimized for MP2RAGE sequences, which achieved a sensitivity of 72.9% and a specificity of 83.0% in the original validation cohort.^7^, ^8^, ^20^ To ensure comparability between volume‐ and surface‐based frameworks, both MAP18 and MELD were applied to T1MPRAGE/3D FLAIR and MP2RAGE sequences from the same MRI acquisition sessions.

### 2.5 Ground truth lesion mask definition

**FIGURE 1:**
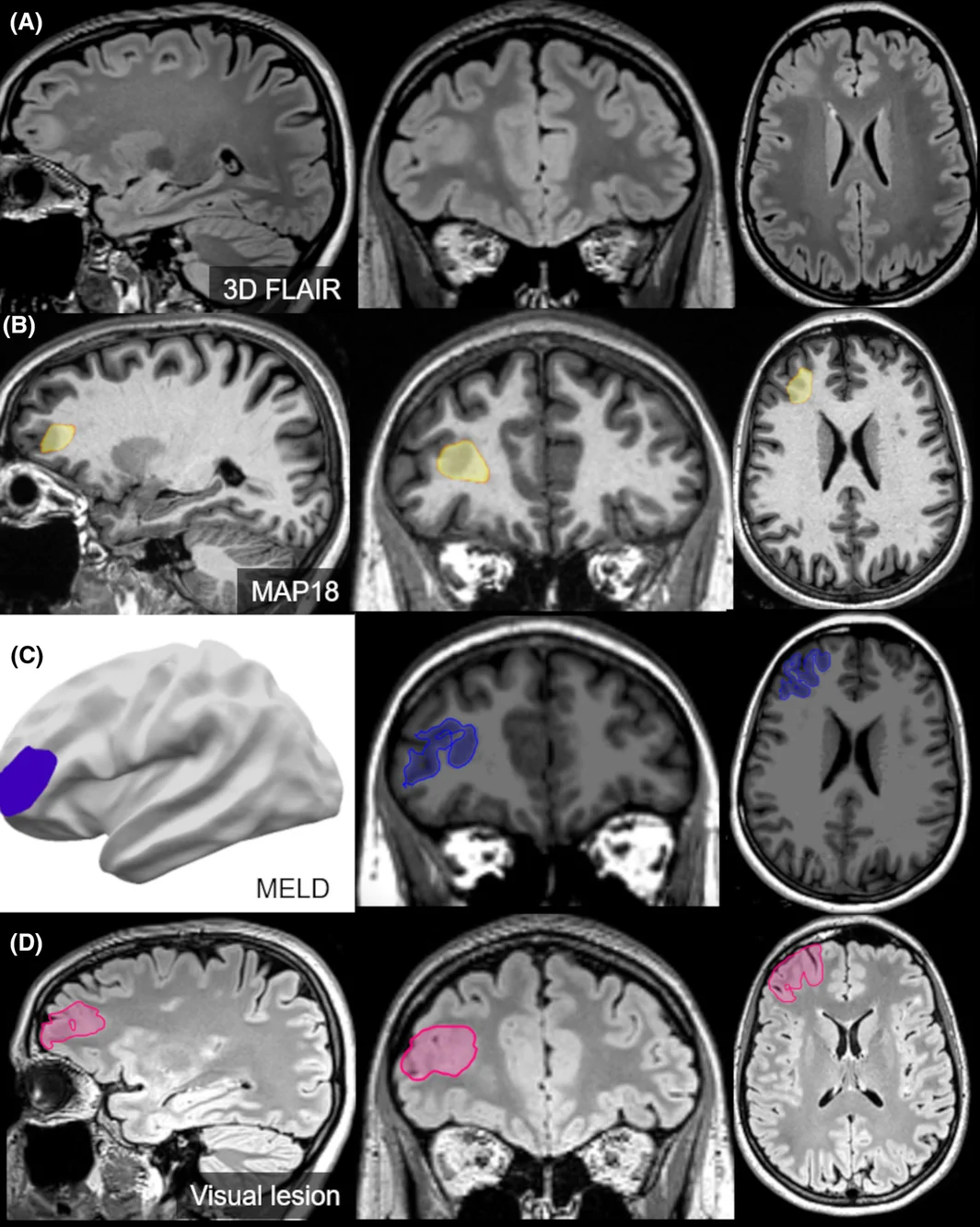
Comparison of automated lesion detection in a patient with focal cortical dysplasia of the right prefrontal lateral cortex illustrated on a (A) three‐dimensional (3D) fluid‐attenuated inversion recovery (FLAIR) sequence. (B) Morphometric Analysis Program, version 2018 (MAP18) output projected onto the magnetization‐prepared 2 rapid gradient echo sequence; a background‐suppressed reconstruction was applied to unify illustration across figures. (C) Multi‐centre Epilepsy Lesion Detection (MELD) Graph algorithm output shown on a reconstructed lateral hemispheric surface and corresponding T1‐weighted planes; true positive cluster is shown in blue. (D) Visually identified lesion (magenta) on 3D FLAIR serving as ground truth. All planar images are shown in sagittal, coronal, and axial orientations unless otherwise indicated.

Ground truth lesion masks for FCD, excluding the subcortical tail in Taylor‐type FCD, were based on established MRI criteria. Lesion regions of interest (ROIs) were delineated using both T1MPRAGE and 3D FLAIR images within the custom imaging platform NORA (https://www.nora‐imaging.com) at the Department of Medical Physics, University Hospital Freiburg. Two independent raters (L.B. and M.H.), blinded to automated detection results, performed visual delineation; discrepancies were resolved by consensus. For pediatric patients, lesion boundaries were verified by a child epileptologist (V.S.A.‐A.). Final binary lesion masks were validated by two experienced neuroradiologists (T.D. and H.U.) and used for subsequent analysis of algorithmic detection performance, as illustrated in Figure 1.

### 2.6 Quantitative performance evaluation

The performance of MELD and MAP18 was evaluated in two ways. First, we compared the detection rates of FCD at the patient level. When clusters of MELD or MAP18 spatially overlapped with the visually drawn ROI, the detection was counted as a true positive. If an FCD existed but was not detected by either method, it was counted as a false negative. In patients where a detection cluster was identified but did not overlap with the known FCD location, this was counted as a false positive. In addition, we evaluated the voxelwise overlap of the detections from all clusters to voxels of the visual FCD ROI.

Several additive metrics were used to assess algorithm performance. The detection rate (overall success rate) was defined as the proportion of patients with at least one true positive cluster overlapping the ground truth mask. Sensitivity (recall) measured the fraction of ground truth lesion clusters successfully detected by each algorithm. Precision was calculated as the ratio of true positive clusters to all detected clusters per patient. The F1‐score, computed as the harmonic mean of precision and sensitivity (F1 = 2 × [precision × sensitivity] / [precision + sensitivity]), provided a balanced assessment of overall performance. Additionally, the false positive rate was determined as the mean number of spurious clusters detected per patient.

### 2.7 Voxelwise comparison

Although cluster‐level detection success was evaluated at the patient level, we also performed a voxelwise overlap analysis to quantify spatial precision and recall. This additional approach was necessary because the clustering strategies of both algorithms produced patterns that complicated direct cluster‐to‐lesion correspondence. The detection algorithms sometimes segmented extended FCDs into multiple discrete clusters based on morphometric discontinuities.

To use objective criteria, we included all detected voxels—including those from clusters distant from the FCD lesion—in the voxelwise comparison. These clustering artifacts necessitated an objective, voxel‐level assessment that accounted for the spatial distribution of all detected voxels, regardless of clustering fragmentation.

For the voxelwise analysis, we calculated recall and precision for each subject by comparing all included detected voxels from both methods against the manually delineated ground truth lesion mask. Statistical comparisons between methods are described in the statistical analysis section.

### 2.8 Clinical correlation analysis

To evaluate the clinical correlates of the detected lesion clusters, spatial correspondence with pre‐ and postoperative clinical markers was examined qualitatively. The clinical markers included the IZ and SOZ as determined by noninvasive presurgical video‐electroencephalographic (EEG) monitoring, and in selected patients, by invasive video‐EEG monitoring. Postsurgical seizure outcome was classified using the modified Engel scale (Engel I–IV), with a postsurgical follow‐up period of at least 1 year. Spatial overlap between algorithmic clusters and these clinical features was assessed qualitatively in all patients. FCDs detected by MELD and/or MAP18 that showed concordance with the IZ or SOZ were considered clinically supported FCD detections.

### 2.9 Statistics

Cluster‐level precision and false positive counts were additionally compared between algorithms using Wilcoxon signed‐rank tests with Bonferroni correction (*α* = .025), with rank‐biserial correlation (rb) reported as effect size where the distribution permitted meaningful interpretation. For voxelwise overlap analysis, we quantified the comparison between visually marked ground truth and automated MELD and MAP18 detections using recall, precision, F1‐score, and false positive rate. Paired differences between methods were evaluated using Wilcoxon signed‐rank tests with Bonferroni correction (*α* = .025 for the primary metrics: recall and precision), and rb was reported as the effect size.

## 3 RESULTS

### 3.1 Study population, clinical characteristics, and lesion localization

We retrospectively analyzed 124 patients who underwent epilepsy surgery with histopathological diagnosis of FCD I, FCD II, or mMCD between January 2018 and April 2024. We excluded 67 patients with temporal pole involvement, 17 lacking MP2RAGE sequences, and eight with prior brain surgery or second resection. Our final study cohort comprised 32 patients with histopathologically confirmed FCD (see Figure S1). The cohort included 15 males and 17 females, with a mean age of 21 years (SD = 13 years, range = 1–53 years). Across these patients, a total of 34 FCD lesions were evaluated, as one patient presented with multiple, spatially distinct lesions within the same hemisphere (see details in Table 1).

The histopathological diagnoses included FCD type II in 31 lesions (91.2%) and mMCD in two lesions (5.8%). Due to surgical sampling limitations, there was no FCD subtype specification in one lesion (2.9%).

Lesion distribution by lobe was as follows: frontal lobe (*n* = 19, 55.9%), parietal lobe (*n* = 2, 5.9%), temporal lobe (*n* = 3, 8.9%), central region (*n* = 5, 14.7%), and multilobar temporo‐occipital involvement (*n* = 5, 14.7%). The median lesion volume based on visual delineation was 5.0 cm^3^ (interquartile range [IQR] = 2.6–13.3 cm^3^).

The median epilepsy duration was 8.5 years (IQR = 2–18.5 years). Presurgical evaluation included noninvasive video‐EEG monitoring in the common presurgical Epilepsy Program of the University Epilepsy Center Freiburg, Freiburg, Germany, and the Epilepsy Center Kork, Kehl‐Kork, Germany for all patients. Invasive EEG monitoring was performed at the Epilepsy Center Freiburg in 11 of 32 patients (34%). Ten patients had invasive stereo‐EEG, and one patient underwent invasive subdural EEG. All patients underwent epilepsy surgery. Detailed patient characteristics are presented in Table 1.

### 3.2 Algorithm detection performance

**FIGURE 2:**
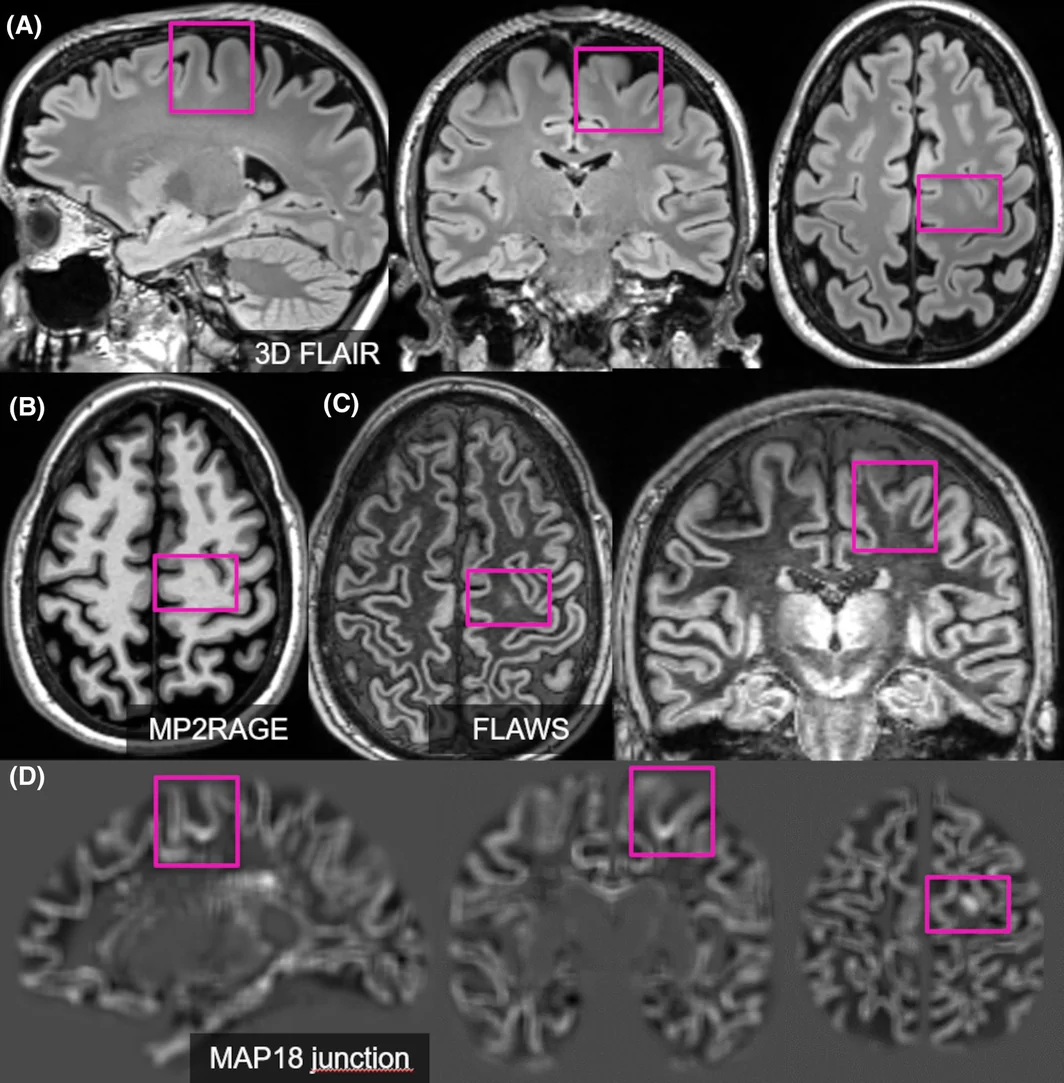
Lesion missed by both magnetic resonance imaging (MRI) morphometry algorithms in a patient with focal cortical dysplasia (FCD) in the left supplementary motor cortex. (A) Three‐dimensional (3D) fluid‐attenuated inversion recovery (FLAIR) sequences are shown in sagittal, coronal, and axial planes. Despite negative automated detection, rigorous visual evaluation of advanced MRI sequences enabled subsequent identification of the FCD, underlining that expert visual assessment of morphometry outputs remains an indispensable part of the diagnostic workup. (B) The axial magnetization‐prepared 2 rapid gradient echo (MP2RAGE) sequence (background‐suppressed reconstruction to unify illustration across figures) demonstrates the subtle lesion, which was negative on automated detection.^25^ (C) Axial and coronal fluid and white matter suppression (FLAWS) sequences^21^ enhanced (D) cortical gray matter junction, and together with junction maps revealed subtle gray–white matter boundary abnormalities, enabling visual identification of the FCD despite negative automated findings. Magenta boxes indicate lesion location. MAP18, Morphometric Analysis Program, version 2018.

**FIGURE 3:**
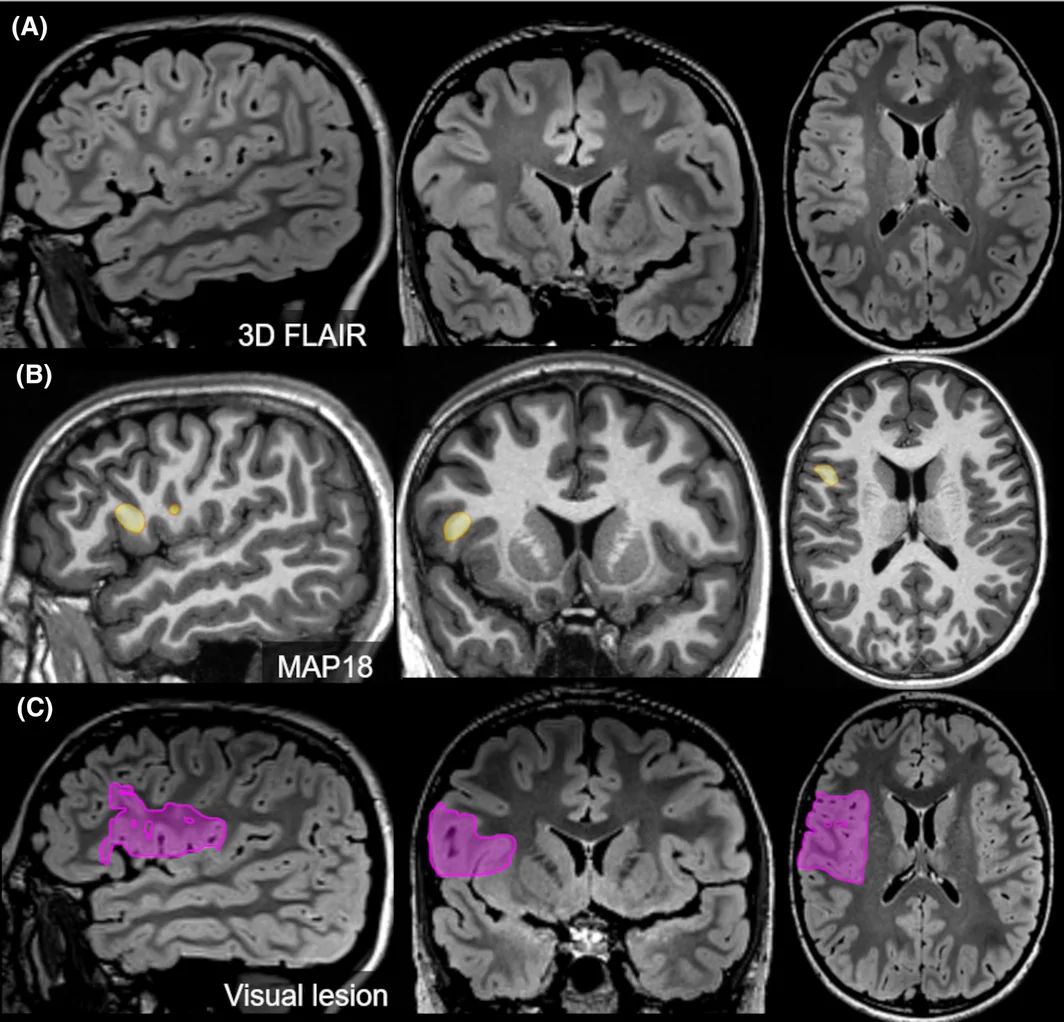
Automated lesion detection in a patient with focal cortical dysplasia (FCD) in the right frontal lobe, correctly identified by Morphometric Analysis Program, version 2018 (MAP18) but not by the Multi‐centre Epilepsy Lesion Detection (MELD) algorithm. (A) Three‐dimensional (3D) fluid‐attenuated inversion recovery (FLAIR), (B) MAP18 cluster projected onto the magnetization‐prepared 2 rapid gradient echo sequence (background‐suppressed reconstruction to unify illustration across figures), and (C) visually identified lesion (magenta) serving as ground truth, all shown in sagittal, coronal, and axial planes. The lesion was correctly detected by MAP18 but missed by the MELD Graph algorithm.

At the lesion level, both MELD and MAP18 detected FCDs with high sensitivity. MELD detected 32 of 34 FCD lesions (94.1%), and MAP18 identified 33 lesions (97.1%). Consensus detection (spatial overlap between both algorithms) was achieved in 32 of 34 lesions (94.1%; Figure 1). The one lesion missed by both algorithms was an FCD type II in the frontal lobe with a volume of .96 cm^3^ (Figure 2). The lesion detected by MAP18 but missed by MELD was an FCD II in the frontal lobe with a volume of 26.95 cm^3^ (Figure 3).

Notably, in the patient with three histopathologically confirmed FCDs, both MELD and MAP18 detected all three lesions (100%), demonstrating robust performance in a multilesion case (Figure S2). At the patient level, MELD identified at least one true positive lesion in 30 of 32 patients (93.8%), and MAP18 achieved detection in 31 of 32 patients (96.8%). Overall patient‐level success is illustrated Figure S3A. Detailed detection performance metrics, including precision, false positive rates, and stratified analyses, are summarized in Table 2.

**TABLE 2:** Detection performance metrics for MELD Graph classifier and MAP18.

Metric	MELD	MAP18
Detection rates		
Lesion level [*n* = 34], *n* (%)	32 (94.1%)	33 (97.1%)
Patient level [*n* = 32], *n* (%)	30 (93.8%)	31 (96.8%)
Cluster metrics per patient (IQR)		
Median clusters per patient	1^a^	4 (2.75–6)
Median true positives per patient	1^a^	1 (1–1)
Median precision	1^a^	.25 (.16–.37)
Median false positives per patient	0^a^	3 (1.75–5)
Performance metrics		
F1‐score	1	.40
Sensitivity	.94	.97
Voxelwise analysis (IQR)		
Median precision	.42 (.25–.78)	.13 (.05–.22)
Median recall	.44 (.27–.71)	.23 (.16–.50)

Abbreviations: IQR, interquartile range; MAP18, Morphometric Analysis Program, version 2018; MELD, Multi‐centre Epilepsy Lesion Detection.

^a^ IQR was not reported for MELD cluster metrics, as the distribution was highly concentrated; 29 of 32 patients had exactly one true positive cluster and precision of 1.0; exceptions are detailed in three patients with false positive detections (mean precision = .92; see Figure S3B,C).

### 3.3 Precision and false positive analysis

MAP18 often identified clusters spatially separated from the detected lesion (two patients (6%) with multiple true positives and additional distant false positives), whereas MELD usually generated only a single cluster. These clustering patterns made one‐to‐one cluster‐to‐lesion correspondence ambiguous, necessitating arbitrary aggregation decisions. We therefore performed voxelwise overlap analysis to provide objective quantitative assessment of spatial precision and recall, accounting for all detected voxels including those distant from the primary lesion.

Cluster‐level analysis demonstrated a tendency for precision and F1‐score to depend on the number of clusters per patient; MELD generated a single cluster in 31 of 32 patients and two clusters in one patient. MELD achieved a median per‐patient precision of 1.0 (IQR = 1.0–1.0); however, false positive clusters were present in three patients: two with no true positive detection (precision = 0) and one with one true positive among two detected clusters (precision = .5), yielding a mean precision of .92. The median cluster confidence score was 84.3% (IQR = 41.3%–95.9%). MAP18 produced a median of four clusters per patient (IQR = 2.75–6, range = 1–19), with a median of one true positive cluster (IQR = 1–1), a mean precision of .25 (IQR = .16–.37), and a median of three false positive clusters (IQR = 1.75–5). The F1‐score was 1.0 for MELD and .40 for MAP18 (Table 2). MELD demonstrated significantly higher cluster‐level precision than MAP18 (Wilcoxon signed‐rank test, *p* < .001, rb = +.98) and significantly fewer false positive clusters per patient (*p* < .001, rb not reported due to near‐complete separation), with a significantly lower total number of clusters overall (*p* < .001).

### 3.4 Voxelwise analysis

Complementing the lesion‐level evaluation, voxelwise analysis was performed to assess spatial precision and recall. MELD demonstrated significantly higher precision compared to MAP18 (Wilcoxon signed‐rank test, *p* = .000014, rb = −.95) as well as a significantly higher recall (*p* = .0057, rb = −.78). MELD achieved a median precision of .42 (IQR = .25–.78) with a median recall of .44 (IQR = .27–.71), whereas MAP18 showed a median precision of .13 (IQR = .05–.22) and a median recall of .23 (IQR = .16–.50). These voxel‐level findings were concordant with the lesion‐level evaluation, where MELD outperformed MAP18 in precision (Figure S4).

### 3.5 Clinical correlation analysis

Spatial concordance between algorithmic detections and established clinical markers of epileptogenicity was evaluated for all patients. There was correct lateralization and lobar‐level concordance with the SOZ defined by EEG in 29 of 32 (90.6%) patients. No definite seizure pattern could be recorded with scalp EEG for the remaining three patients. For the IZ, lobar concordance was observed in 27 of 32 (87.5%) patients. In two patients without lobar concordance, there was still correct lateralization to an adjacent lobe. In the subset of 11 patients who underwent invasive EEG monitoring, clusters overlapped with the SOZ and the IZ defined by invasive EEG in all patients (*n* = 11, 100%). Twenty‐eight of 32 patients (87.5%) achieved seizure freedom (Engel I) with a follow‐up period of more than 12 months. The median follow‐up time was 25 months (SD = 14 months). One additional patient was seizure‐free, but the follow‐up period was less than 12 months. For three patients, seizures persisted after epilepsy surgery due to incomplete resection of the structural lesion. Importantly, algorithmic detection was successful in all three patients; seizure persistence was therefore attributable to incomplete lesionectomy rather than failure of automated detection.

## 4 DISCUSSION

### 4.1 Main findings

Both MELD and MAP18 achieved high detection rates for FCDs in the cohort of 32 epilepsy patients with histopathologically proven FCDs, with strong concordance between methods (97.1% of lesions detected by both algorithms). The algorithms demonstrated lesion‐level sensitivities of 94.1% (MELD) and 97.1% (MAP18), as well as patient‐level sensitivities of 93.8% (MELD) and 96.8% (MAP18). These results exceed previously published single‐center MAP18 results of 72.9% on MP2RAGE sequences^10^ as well as the MELD Graph multicenter validation report with a sensitivity of 72% and PPV of 76% in a site‐independent cohort.^10^ Both algorithms missed a single frontal FCD type II (.96 cm^3^), and MAP18 detected one frontal FCD II (26.95 cm^3^) that was missed by MELD (Figures 2 and 3). The failure of MELD Graph to detect this large lesion may reflect the diffuse cortical surface signature of extensive FCDs, which can lack the focal morphometric contrast that surface‐based classifiers rely on. Taking this near‐concordance into account, the overall performance of MELD and MAP18 was nearly equivalent at the patient level. The overall superior performance of both morphometry methods compared to prior reports likely reflects the homogeneous, high‐resolution 3‐T imaging cohort from a single scanner with a standardized protocol, including T1MPRAGE, 3D FLAIR, and MP2RAGE sequences with isotropic voxels of 1‐mm edge length, as opposed to heterogeneous multicenter validation studies employing mixed 1.5‐T and 3‐T MRI protocols with variable acquisition parameters.^15^, ^19^, ^22^, ^23^ Additionally, these findings were supported by clinical validation, as both algorithmic detections showed high concordance with SOZ and IZ from invasive and scalp EEG. Lobar‐level concordance reached 90.6% for the SOZ and 87.5% for the IZ in scalp EEG. The SOZ and IZ were confirmed in the area of the FCD in all 11 patients with invasive EEG.

### 4.2 Methodological differences between MELD and MAP18

Both MRI morphometry algorithms use entirely different approaches; MELD integrates cortical surface reconstruction, whereas MAP18 is a volume‐based postprocessing tool. MELD employs a surface‐based approach, utilizing FreeSurfer v7.2 surface reconstructions with deep learning on 34 vertexwise features including cortical thickness, curvature, and FLAIR intensity.^10^ MAP18 is implemented within the SPM12 framework and produces three standardized morphometric contrasts. MP2RAGE sequences were processed in place of T1MPRAGE, as their superior B1 field homogeneity yields clearer morphometric maps with larger lesion volumes and higher statistical *z*‐scores.^8^ Thus, subtle, small, or cortical–subcortical junction abnormalities that might be overlooked on conventional sequences are highlighted. The large lesion missed entirely by MELD Graph (26.95 cm^3^) but only partially detected by MAP18 illustrates the complementary limitations of both approaches for extensive FCDs; surface‐based morphometry may lack sensitivity to subcortical and transmantle components, and MAP18's volumetric approach, despite detecting parts of the lesion, did not achieve complete detection.

Despite comparable sensitivities at the lesion level, voxelwise analysis revealed that MAP18 demonstrated significantly lower precision compared to MELD. This reflects MAP18's volume‐based approach, which detects subcortical white matter changes associated with FCD, including transmantle signs and other pathological features,^23^ that MELD cannot identify because it restricts analysis to cortical surface regions. Conversely, MELD Graph's surface‐confined architecture resulted in significantly fewer false‐positive clusters per patient, which is an important specificity advantage for clinical implementation. Because MAP18's neural network was originally trained on T1MPRAGE rather than MP2RAGE data, retraining on MP2RAGE‐specific intensity profiles could further enhance its detection performance.^8^ Although these subcortical detections represent genuine pathological findings, they extend beyond visually delineated cortical lesion masks, resulting in reduced voxel‐level precision. Thus, MAP18's broader spatial detection captures clinically relevant gray matter extensions and transmantle dysplasia patterns, although at the cost of reduced precision when evaluated against cortical‐focused visually delineated lesion ROIs. Given that morphometry algorithms may be insufficient for precisely delimiting lesion extent in extended FCDs, especially type 1 FCDs and mMCDs, stereo‐EEG remains essential in clinical practice for accurately defining resection boundaries in challenging cases.^24^ Given that morphometry algorithms may be insufficient for precisely delimiting lesion extent in extended FCDs, especially type I FCDs and mMCDs, stereo‐EEG remains essential in clinical practice for accurately defining resection boundaries in challenging patients.^25^

### 4.3 Multimodal integration

The present study employed morphometric features derived from T1MPRAGE, MP2RAGE, and 3D FLAIR sequences. Emerging evidence suggests that incorporating additional quantitative MRI parameters, including relaxation time mapping, diffusion tensor imaging, and magnetic resonance fingerprinting, could provide complementary pathophysiological information. Recent studies combining quantitative parameters with MAP18 have demonstrated value in reducing false positive detections while maintaining sensitivity.^26^, ^27^

Beyond multimodal MRI integration, combining automated lesion detection with source imaging and electrophysiological markers enhances clinical utility. In the present study, algorithmic detections demonstrated good lobar concordance with SOZ and IZ from scalp EEG. Combined MRI morphometry and source imaging can guide stereo‐EEG electrode placement to delimit the epileptogenic zone in cases with subtle or absent lesions.^25^, ^28^, ^29^ Concordance between source imaging and morphometric findings relates to favorable postsurgical outcomes.^30^ Integration with fluorodeoxyglucose positron emission tomography further improves FCD detection.^31^ Several intracranial EEG parameters may additionally contribute to better delineating the spatial extent of subtle FCDs, including interictal fast oscillations and their spectral bandwidth,^32^ fast oscillations increasing during sleep,^33^ gamma oscillation regularity,^34^ and gamma amplitude–envelope correlations within hyperexcitable networks.^35^ Noninvasive assessment of fast oscillations using sleep magnetoencephalography may further extend this approach.^36^ Such multimodal approaches may be particularly valuable for patients with multiple potentially epileptogenic lesions or ambiguous structural findings, providing complementary information for surgical planning.

### 4.4 Limitations

Several limitations warrant consideration. First, our single‐center design using standardized protocols, while ensuring homogenous, high‐quality imaging, limits generalizability to centers with heterogeneous imaging equipment. Second, exclusion of temporal pole lesions resulted in a cohort enriched for extratemporal FCD type II (91.2%), and inclusion was restricted to surgically treated patients, who typically have electroclinical concordance, introducing selection bias toward MRI‐visible lesions with higher detection rates than unselected cohorts. Third, visual delineation focused on cortical abnormalities and excluded subcortical components, disadvantaging MAP18's transmantle detection in precision metrics even when identifying genuine pathology. Finally, clinical validation was descriptive; although algorithmic detections showed high lobar concordance with SOZ and IZ, we did not formally test whether detected regions overlap with resection zones or predict surgical outcomes.

### 4.5 Clinical implementation

Successful clinical implementation of automated FCD detection requires addressing practical challenges beyond algorithmic performance metrics. The present single‐center study with standardized protocols achieved high detection rates (93%–97%), contrasting with multicenter evaluations that reveal substantial performance variability across imaging environments, with detection rates ranging from 33% to 57% depending on protocol standardization.^19^ These findings highlight two complementary challenges: performance variability across multicenter environments due to heterogeneous imaging protocols, and variability even within single centers using 3‐T MRI, as recently demonstrated.^18^ Further prospective multicenter studies with harmonized acquisition protocols are needed to determine the conditions under which reliable clinical implementation can be achieved. Integration into diagnostic workflows must balance sensitivity with specificity, as automated detection serves most effectively as a decision‐support tool guiding clinician review rather than replacing clinical judgment.^22^ Practical requirements include reasonable computational runtime for workflow integration, software compatibility with hospital systems, and clinician‐friendly interpretability with confidence scores. For the MP2RAGE‐based MAP18 algorithm, we developed an automated pipeline that stores inversely normalized overlays of MAP18 lesions on the MP2RAGE sequence in the Picture Archiving and Communication System (PACS), thus allowing easy identification of subtle, otherwise overlooked FCD lesions.^37^ Future development should focus on balancing sensitivity–precision trade‐offs while maintaining acceptable runtime, and prospective studies determining how algorithm‐guided workflows improve diagnostic accuracy and surgical outcomes across diverse clinical settings.

## AUTHOR CONTRIBUTIONS

Lara Bücheler collected clinical data, performed ROI marking, conducted image and initial statistical analysis, and wrote the first draft of the manuscript, all under the supervision of Marcel Heers. Marco Reisert contributed multimodal image registration and statistical analysis. Theo Demerath, Victoria San Antonio‐Arce, Horst Urbach, and Marcel Heers performed MRI evaluation and ROI correction. Florian Mayer contributed to clinical data collection and supervised its acquisition. Christian Moeller and Joshua Reß supported implementation of the morphometry and imaging pipelines. Yiwen Li Hegner contributed to multimodal characterization of the cohort within the framework of the overarching research program. Dirk‐Matthias Altenmüller contributed to clinical characterization of the cohort. Christian Scheiwe and Mukesch Shah evaluated results from a neurosurgical perspective and performed the resections. Soroush Doostkam evaluated histopathological specimens. Jürgen Beck, Thomas Bast, Bernhard J. Steinhoff, and Andreas Schulze‐Bonhage facilitated patient recruitment and evaluated clinical aspects. Horst Urbach coconceived the study, cosupervised the first author, and contributed to manuscript writing and figure design. Marcel Heers conceived and designed the study, supervised the first author, wrote the manuscript, and is responsible for the overall integrity of the work. Horst Urbach and Marcel Heers contributed equally as senior authors. All authors critically appraised the results, reviewed the manuscript, and approved its final version.

## FUNDING INFORMATION

Y.L.H. (LI1904/2‐1) and M.H. (HE6844/3‐1; project ID for both: 468174690) were funded by the German Research Foundation.

## CONFLICT OF INTEREST STATEMENT

T.D. is a consultant for Medtronic; and has received travel & educational grants from Balt, Stryker, and Medtronic. A.S.‐B. has received research support from BIAL, Precisis, and UNEEG, and personal honoraria for lectures or advice from Angelini Pharma, Jazz/GW Pharmaceuticals, Precisis, UCB, and UNEEG. B.J.S. has received advisory and consulting honoraria from Angelini, Jazz/GW Pharmaceuticals, Neuraxpharm, Precisis, Roche Diagnostics, and UCB; has received speaker's honoraria from Al Jazeera, Angelini, BIAL, Desitin, Eisai, Jazz/GW Pharmaceuticals, Medscape, Tabuk, Teva, UCB, and Zogenix; and has received research support from Eisai, the European Union, Jannsen‐Cilag, Jazz/GW Pharmaceuticals, SK Life Sciences, UCB, and Zogenix. H.U. has received honoraria for lectures from Biogen, Eisai, Mbits, Lilly, and Bayer; is supported by German Federal Ministry of Education and Research; and is coeditor of *Clinical Neuroradiology*. M.H. has received support for conference participation from GW Pharmaceuticals and Precisis, and speaker's honoraria for lectures from Eisai, GW Pharmaceuticals, and Arkana. None of the other authors has any conflict of interest to disclose. We confirm that we have read the Journal's position on issues involved in ethical publication and affirm that this report is consistent with those guidelines.

## Supporting information


Figure S1.

## Data Availability

The data that support the findings of this study are available on request from the corresponding author. The data are not publicly available due to privacy or ethical restrictions.
