# Supplementary material for: Comparison of volume‐ and surface‐based magnetic resonance imaging morphometry algorithms in the detection of focal cortical dysplasia
Source: Epilepsia. 2026 Jun 3;67(9):4662–74. doi: 10.1002/epi.70326 (PMC13592473; doi:10.1002/epi.70326)
Supplement: Supplementary file 1 [file EPI-67-4662-s001.pdf]

Supplementary Files:
Comparison of Volume- and Surface-based MRI Morphometry Algorithms in the Detection of Focal Cortical Dysplasia

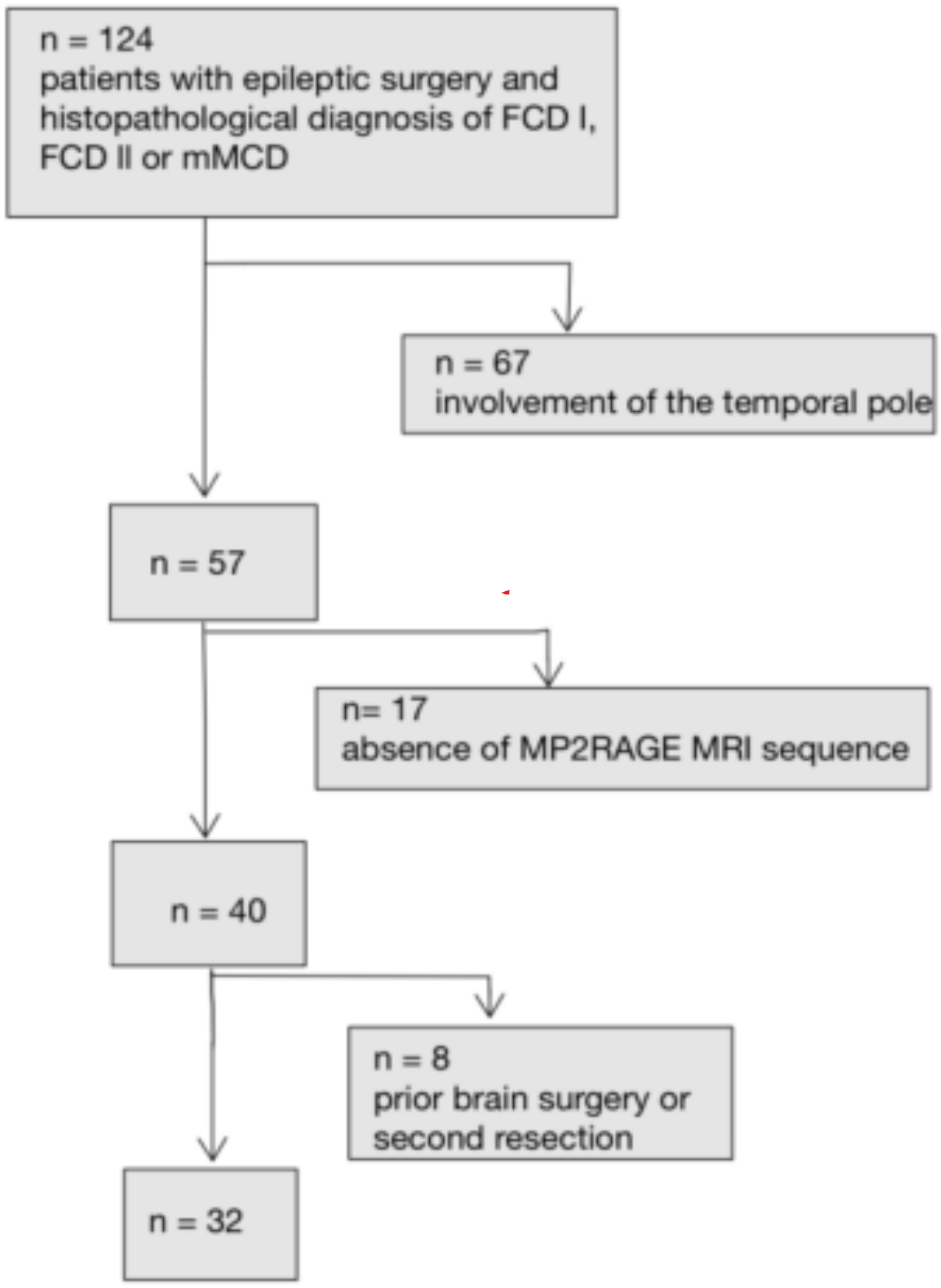

Suppl. Fig. 1. CONSORT Chart of Patient Inclusion. This figure outlines the process of patient selection and inclusion for the study cohort (32 patients, 34 lesions). The chart illustrates the steps leading to the final study population, including eligibility criteria, exclusions, and confirmation of focal cortical dysplasia (FCD) by both MRI and histopathology. Abbreviations: mMCD, mild malformation of cortical development; MP2RAGE, Magnetization Prepared 2 Rapid Gradient Echo.

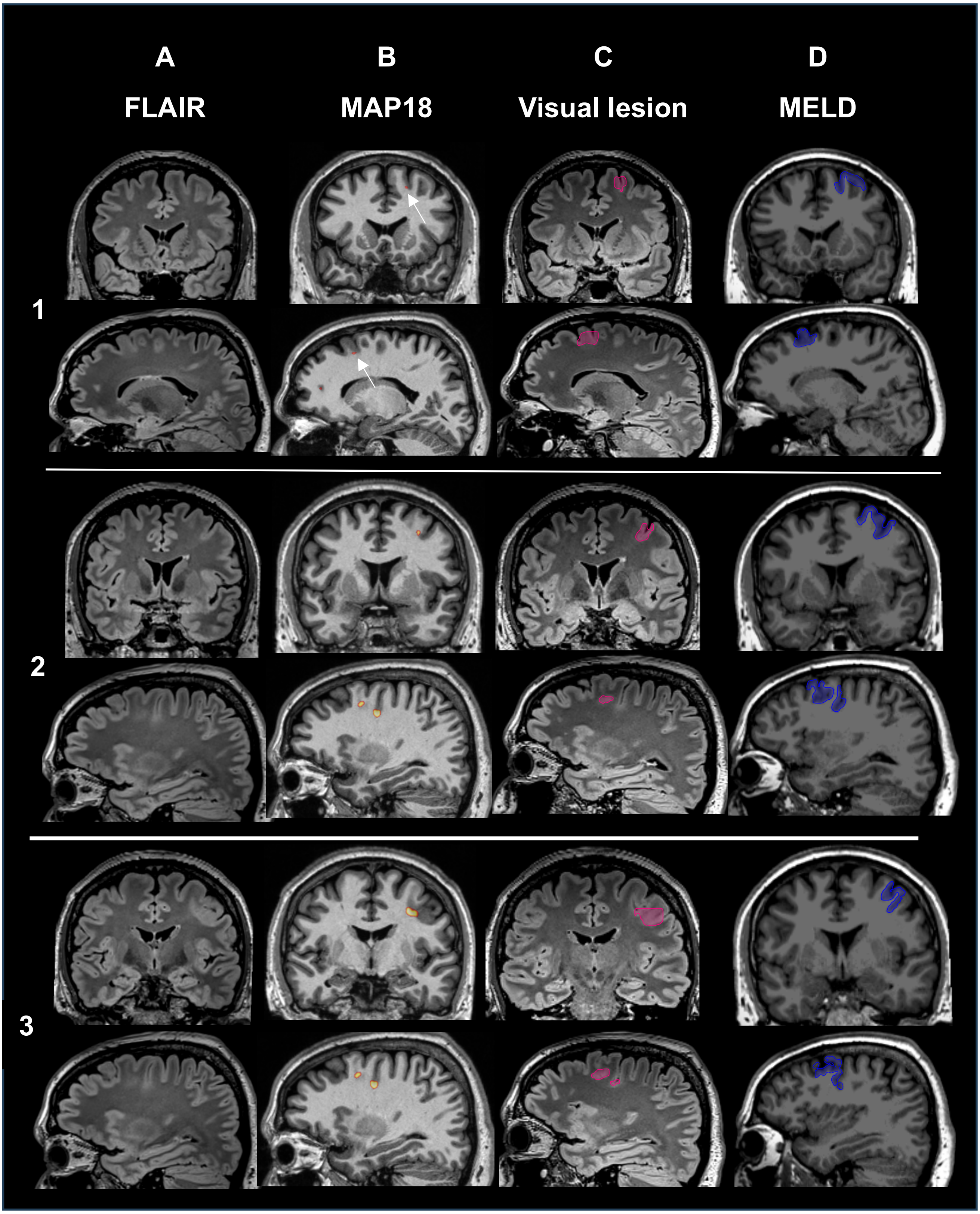

Suppl. Fig. 2. Automated lesion detection in a patient with three histopathologically confirmed focal cortical dysplasias (FCDs) in the left frontal lobe. Lesions are located in the superior frontal sulcus (row 1), middle frontal sulcus (row 2), and precentral sulcus (row 3), shown in coronal and sagittal planes. Columns show 3D fluid-attenuated inversion recovery (FLAIR; A), Morphometric Analysis Program (MAP18; B), visually identified lesions serving as ground truth (magenta; C), and Multi-centre Epilepsy Lesion Detection (MELD; D). MAP18 detected all three lesions as distinct clusters, whereas MELD identified them as a single extended cluster spanning all three regions.

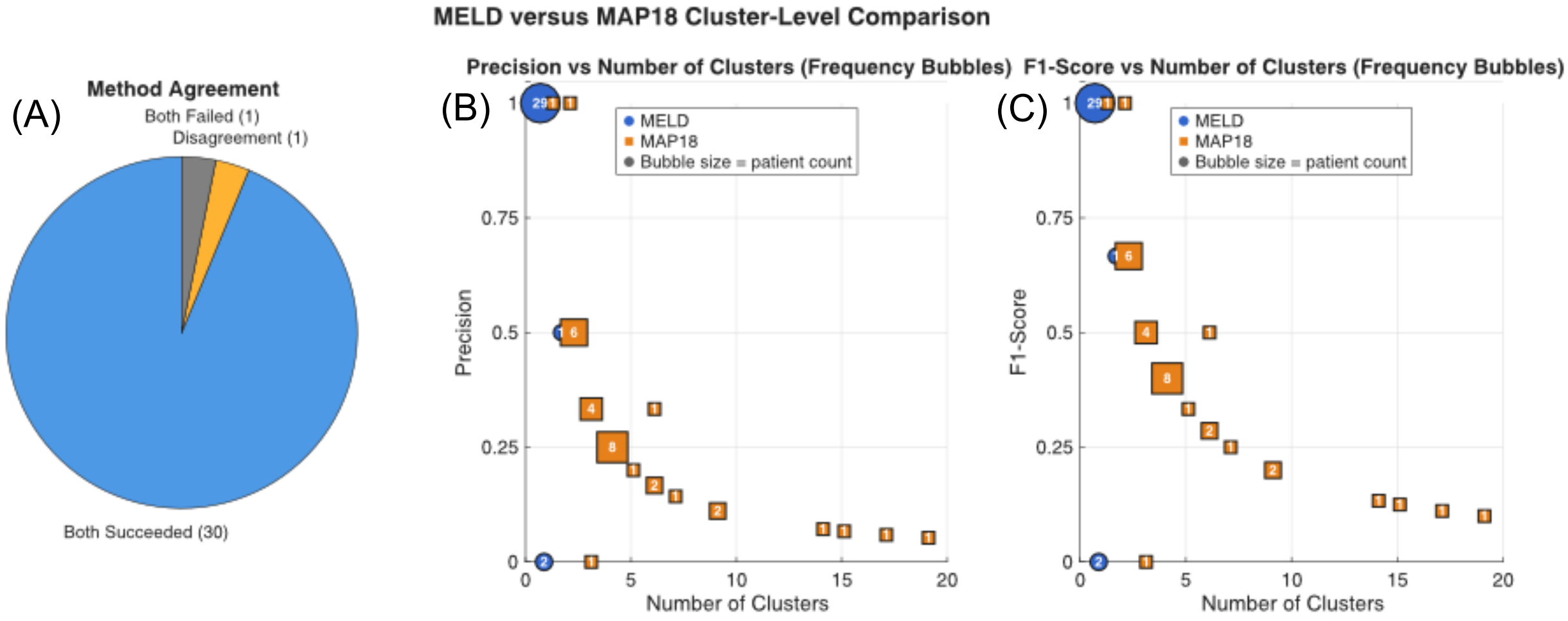

Suppl. Fig. 3: Patient-level detection performance and influence of cluster number on precision and F1-score for MELD and MAP18. Analysis was performed at the patient level; the patient with three histopathologically confirmed FCDs was treated as a single detection unit. Pie chart showing method agreement: both algorithms successfully detected lesions in 30 patients, both failed in 1 patient, and there was disagreement in 1 patient. (B) Precision and (C) F1-score versus number of clusters per patient for MELD and MAP18; bubble size reflects patient count per cluster-number category. Both metrics decline with increasing cluster number for both algorithms.

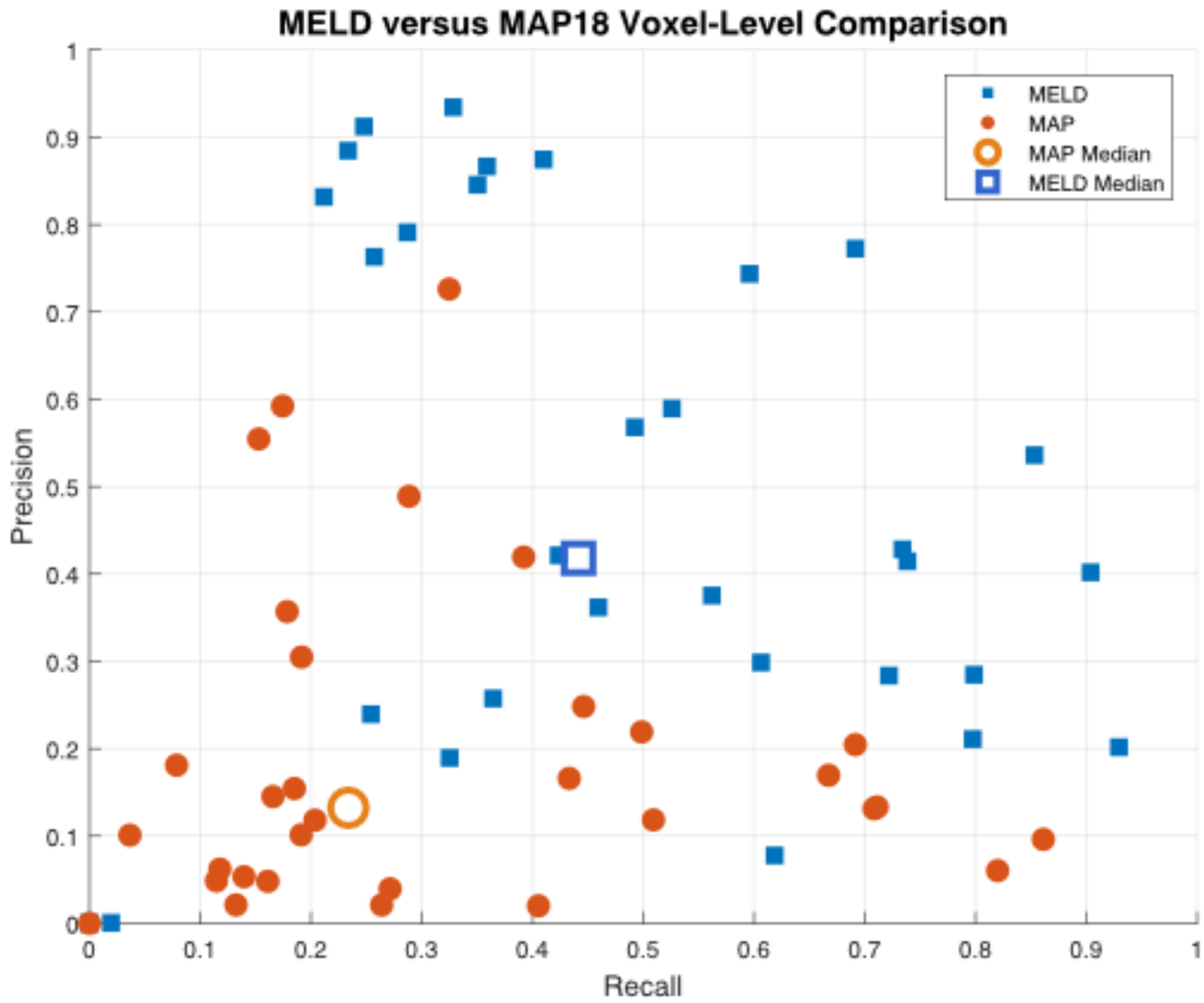

Suppl. Fig. 4: Precision versus recall comparison at the voxel-level between MAP18 and MELD algorithms. Scatterplot of precision versus recall (sensitivity) for MAP18 (orange circles) and MELD (blue squares) across all patients. Each point represents the performance for an individual patient. Median values are indicated by larger hollow symbols (MAP18 median: recall=0.23, precision=0.13; MELD median: recall=0.22, precision=0.40). MELD demonstrates higher precision compared to MAP18 at comparable recall values, indicating fewer false positive detections.
